# Phylogenetic Analysis of a Human Isolate from the 2000 Israel *West Nile virus* Epidemic

**DOI:** 10.3201/eid0805.010324

**Published:** 2002-05

**Authors:** Thomas Briese, Andrew Rambaut, Melissa Pathmajeyan, Jihad Bishara, Miriam Weinberger, Silvio Pitlik, W. Ian Lipkin

**Affiliations:** * University of California at Irvine, Irvine, California, USA; † University of Oxford, Oxford, UK; ‡ Rabin Medical Center–Beilinson Campus, Petah Tikva, Israel

**Keywords:** *West Nile virus*, WNV, encephalitis, Israel, polymerase chain reaction, PCR, brain tissue, phylogenetic analysis

## Abstract

Specimens from a patient of the 2000 Israel *West Nile virus* epidemic were analyzed by reverse transcription-polymerase chain reaction. Products corresponding to E, NS3, and NS5 sequences were amplified from cerebellar but not from cortical samples. Phylogenetic analyses indicated a closer relationship of this isolate to 1996 Romanian and 1999 Russian than to 1998-99 Israeli or 1999 New York isolates.

West Nile fever is typically a mild febrile illness characterized by headache, myalgias, lymphadenopathy, and a maculopapular rash; West Nile fever occurs sporadically throughout endemic areas of northeastern Africa and tropical Asia (*1*–*5*). The causative agent, *West Nile virus* (WNV), is a member of the genus *Flavivirus* (family *Flaviviridae*), which is grouped together with Alfuy virus, *Cacipacore virus*, *Koutango virus*, *Japanese encephalitis virus*, Kunjin virus, *Murray Valley encephalitis virus*, *St. Louis encephalitis virus*, *Usutu virus*, and *Yaounde* *virus* in the *Japanese encephalitis virus* antigenic complex (*6*,*7*). Two different lineages (I and II) of WNV are characterized genetically (*8*). Whereas lineage II viruses are isolated in endemic areas, lineage I viruses are isolated during epidemic outbreaks of WNV infection and may cause severe encephalitis in the elderly or immunosuppressed persons. Epidemics of West Nile fever were first reported in the 1950s in Israel (*2*) and the 1970s in South Africa (*9*). Sites of notable recent outbreaks include Romania (1996, 1997), Italy (1998), Russia (1999), France (2000), United States (1999, 2000) and Israel (2000) (*5*,*10*). Sequence analysis of the WNV responsible for the United States outbreak in 1999 (WNV-NY1999) showed a close phylogenetic relationship to a WNV isolated from a goose in Israel in 1998 (WNV-ISR1998) (*11*,*12*).

WNV is transmitted mainly by mosquito vectors, although it has also been isolated in several tick species (*3*,*5*,*13*). Birds are an important WNV reservoir. In several avian species, virus replication generates serum titers sufficient to sustain arthropod transmission (*4*,*5*). Birds, during seasonal migrations, are also believed to be instrumental in the geographic spread of WNV (*3*–*5*,*14*). The virus is only occasionally transmitted to humans or other mammals. Viremia in mammals is low level; thus, mammals are considered to be dead-end hosts.

## The Study

From July through November 2000, a WNV epidemic occurred in central and northern Israel. More than 430 people were diagnosed with WNV infection; 29 of these patients had fatal encephalitis. We report phylogenetic analysis of WNV sequences isolated from the brain of an encephalitis patient from the 2000 Israel epidemic.

A 72-year-old woman with a history of recurrent meningioma of the sphenoidal ridge, dementia, and depression was hospitalized because of fever and general deterioration of 5 days’ duration. On admission, the patient was responsive only to painful stimuli and had generalized muscle stiffness and limb tremors. Clinical and laboratory values were consistent with viral encephalitis; thus, the patient was initially treated with intravenous acyclovir for presumptive herpes simplex encephalitis. When polymerase chain reaction (PCR) analysis of cerebrospinal fluid (CSF) showed no evidence of herpes simplex virus infection, and WNV antibodies were detected in serum and CSF, acyclovir was discontinued and ribavirin was initiated at an oral dosage of 2.4 g per day. The patient’s clinical status continued to deteriorate with aspiration pneumonia and intermittent generalized seizures. Intravenous immunoglobulin was added (35 g/d for 2 days) without improvement. Approximately one month after onset of illness, the patient died of respiratory failure.

Postmortem examination of the brain showed multiple meningiomas, generalized atrophy, and surgical resection of the right parietal lobe. Histology was remarkable for neurofibrillary plaques consistent with Alzheimer’s disease, and scattered microglial nodules and perivascular lymphocytic inflammation in the medulla, pons, and midbrain were consistent with viral encephalitis.

RNA was extracted from frontal cortex and cerebellum with TRI-Reagent (Molecular Research Center, Cincinnati, OH). Four micrograms of total RNA from each brain region was used as a template for reverse transcription-polymerase chain reaction (RT-PCR) with primer sets representing three regions of sequence conservation in flavivirus genomes: NS3-1 (EDL/Fla-U5004, 5´- GGA ACD TCM GGH TCN CCH AT and EDL/Fla-L5457, 5´- GTG AAR TGD GCY TCR TCC AT), NS5-1.1 (EDL/Fla-U9093, 5´- AGY MGR GCH ATH TGG TWY ATG TGG and EDL/Fla-L9279, 5´- TCC CAV CCD GCK GTR TCA TC), and NS5-2 (EDL/Fla-U9954, 5´- GSS AAA KCH TAY GCN CAV ATG TGG and EDL/Fla-L10098 5´- AGC ATR TCT TCH GTN GTC ATC CA) (*15*,*16*). Amplification products were obtained with RNA derived from the cerebellum in reactions with all three primer sets; no amplification products were obtained with RNA from the cortex. These amplification products were cloned into the pGEM-Teasy vector (Promega, Madison, WI) and subjected to automated dideoxy sequencing (ABI Prism Model 377, Foster City, CA)*.* Sequences were submitted to GenBank (NS3, GenBank accession no. AF394218; NS5, GenBank accession nos. AF394219 and AF394220).

Signal of cerebellar amplification products in ethidium bromide-stained gels was reduced in comparison with similar studies performed with brain materials from four patients of the 1999 New York City outbreak (data not shown; New York patients were 75 years to 80 years of age, 3 male, 1 female, who died of severe WNV encephalitis during the 1999 outbreak [16]). The relative virus load was 140 copies/200 ng RNA in the Israeli sample, indicated by 5´-nuclease real-time RT-PCR (17), compared with 7000 to 20 copies/200 ng RNA in specimens analyzed from the New York City outbreak (Table). However, since the virus load of the sample from Israel was within the range of virus loads observed with the New York samples, this result for a single Israeli sample may not indicate a strain difference. Quantitative analysis was restricted to the NS5 target because no signal was obtained with primer/probe set prNS3 (fwd, 5´- GCa CTG AGA GGA CTG CCc AT; probe, 5´-6FAM-TAc CAG ACA TCc GCA GTG cCC AGA-T-TAMRA; rev, 5´- TGg GTG AGG GTa GCA TGa CA), because of point mutations in WNV-ISR2000 sequence that prevented efficient hybridization with the primer and probe oligonucleotides (fwd - 2 mismatches, probe – 3 mismatches, rev - 3 mismatches; given above in lower case). Sensitivity was not substantially reduced in assays with primer/probe set prNS5, which had one mismatch in the 3′-terminal sequence of the probe oligonucleotide (Table).

**Table:** Real Time reverse transcription-polymerase chain reaction (RT-PCR) analysis of RNA extracts from 2000 Israel West Nile patient specimens using primer set NY1999-NS5

NS5 standard NY1999		Armored RNA		Armored RNA extract		NY1999 specimens			NS5 standard ISR2000		ISR2000 specimens		
Amount^a^	C_T_^b^	Dil.^c d^	Amount	Dil.^e^	Amount^d^	Patient no.	C_T_	Amount	Amount^f^	C_T_	Sample	C_T_	Amount^g^
2.5x10^6^	16.4	1:10^1^	4.5x10^6^	1:10^1^	4.4x10^5^	1	29.3	6.9x10^2^	2.5x10^6^	16.5	cereb.	31.1	1.4x10^2^
2.5x10^5^	20.0	1:10^2^	3.4x10^5^	1:10^2^	4.0x10^4^	2	25.6	7.3x10^3^	2.5x10^5^	20.0	cortex	36.4^h^	1.9x10^0^
2.5x10^4^	23.6	1:10^3^	3.1x10^4^	1:10^3^	4.3x10^3^	3	30.0	4.6x10^2^	2.5x10^4^	23.1			
2.5x10^3^	27.2	1:10^4^	3.1x10^3^	1:10^4^	6.0x10^2^	5	34.8	2.3 x10^1^	2.5x10^3^	26.7			
2.5x10^2^	31.1	1:10^5^	3.4x10^2^	1:10^5^	9.8x10^1^				2.5x10^2^	30.3			
2.5x10^1^	34.9	1:10^6^	3.1x10^1^	1:10	2.1x10^0^				2.5x10^1^	34.1			
2.5x10^0^	36.8^h^	1:10^7^	3.0x10^0 h^	1:10^7^	n.d^i^				2.5x10^0^	37.3 ^h^			
0	>45	0	0	0	0				0	>45			

^a^ Plasmid DNA p88-D-21 was quantitated spectrophotometrically, and dilutions containing the indicated copy number of target sequence were added to each polymerase chain reaction (PCR) assay. ^b^ C_T_ , Cycle number at which signal crosses threshold. ^c^ Armored RNA *West Nile virus* (HNY1999) standard (Ambion, Austin, TX) was diluted 1:10, boiled, reverse transcribed, and then diluted to result in amounts per PCR assay equivalent to the indicated dilution of the stock (5 μL). ^d^ Amount calculated based on calibration curve obtained with NS5 Standard NY1999 (column 1). ^e^ Dilutions of Armored RNA *West Nile virus* (HNY1999) standard (Ambion) were extracted with TRI-Reagent (Molecular Research Center, Cincinnati, OH) and then subjected to RT-PCR to result in amounts per assay equivalent to the indicated dilution of the stock (5 μL). ^f^ Plasmid DNA pISR-Dfrag-D6 was quantitated spectrophotometrically, and dilutions containing the indicated copy number of target sequence were added to each PCR assay. ^g^ Amount calculated based on calibration curve obtained with NS5 Standard ISR2000 (column 6). ^h^ Poisson effects take place at low template concentration; duplicate assay deviations: 36.2 / 37.4, NY1999; 6.0 x10^0^ / 0, armored RNA; 37.4 / 37.1, ISR2000; and 36.4 / >45, cortex. ^i^ n.d. = not determined.

Sequence analysis of the cloned NS3 and NS5 gene fragments indicated similarity to completely sequenced Romanian and Russian WNV isolates WNV-RO97-50-1996 and WNV-RUS-VLG4-1999, respectively; thus, to facilitate detailed phylogenetic analysis, E gene sequence from the Israel human brain sample was amplified. An E gene sequence of 1509 nucleotides (GenBank Accession Number AF394217) was amplified from total RNA by using GeneChoice UNIPOL polymerase (PGC Scientific, Gaithersburg, MD) and primers EDL/E-U1006 (5´- GGA GTG TCT GGA GCA ACA TGG GT) or EDL/E-U1476 (5´- TCC TGC GGC GCC TTC AT) and EDL/E-L2244 (5´- CCC CTC CAA CTG ATC CAA AGT CC) or EDL/E-L2538 (5´- TCC ATC CAA GCC TCC ACA TCA), respectively. Sequence analysis of this fragment confirmed data from NS3 and NS5 sequence analyses, indicating a closer relationship of WNV-ISR-hISR2000 sequence to Romanian and Russian isolates than to the 1997/98/99 Israeli and the WNV-NY1999 isolates (Figure).

**Figure:**
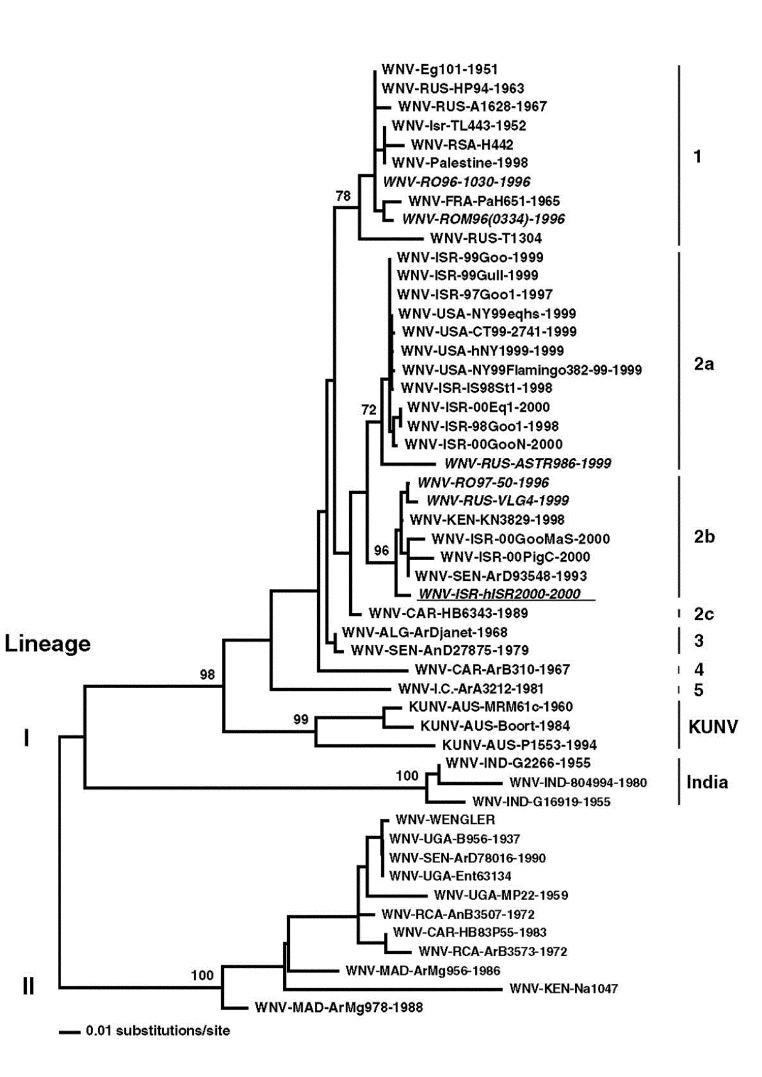
Phylogenetic analysis of WNV-hISR2000 E gene sequence. Phylogenetic analysis of the sequences listed below was performed with PAUP (Phylogenetic analysis using parsimony) 4.0b8 (Sinaur Associates, Sunderland, MA). A neighbor-joining tree was constructed using maximum likelihood distances with the HKY85 model of substitution and allowing different rates of substitution at each codon position. Bootstrap values are the result of 1000 neighbor-joining replicates under this same model. Only relevant bootstrap values are shown. WNV-Eg101-1951 (human, H), AF260968; WNV-RUS-HP94-1963, AF237565; WNV-RUS-A1628-1967 (bird, B), AF237563; WNV-ISR-TL443-1952 (H), AF205881; WNV-RSA-H442, AF205880; WNV-Palestine-1998, V. Deubel unpub.data; WNV-RO96-1030-1996 (H), AF130363; WNV-FRA-PaH651-1965 (H), AF001560; WNV-ROM96(0334)-1996, AF208579; WNV-RUS-T1304; AF237566; WNV-ISR-99Goo-1999 (B), AY033391; WNV-ISR-99Gull-1999 (B), AY033390; WNV-ISR-97Goo1-1997 (B), AF380663; WNV-USA-NY99eqhs-1999 (equus, E), AF260967; WNV-USA-CT99-2741-1999 (mosquito, M), AF206518; WNV-USA-hNY1999-1999 (H), AF202541; WNV-USA-NY99Flamingo382-99-1999 (B), AF196835; WNV-ISR-IS98ST1-1998 (B), AY033389; WNV-ISR-00Eq1-2000 (E), AF380669; WNV-ISR-98Goo1-1998 (B), AF205882; WNV-ISR-00GooN-2000 (B), AF380665; WNV-RUS-ASTR986-1999 (H), AF237562; WNV-RO97-50-1996 (M), AF260969; WNV-RUS-VLG4-1999 (H), AF317203; WNV-KEN-KN3829-1998 (M), AF146082; WNV-ISR-00GooMaS-2000 (B), AF380667; WNV-ISR-00PigC-2000 (pig, P); WNV-SEN-ArD93548-1993 (M), AF001570; WNV-ISR-hISR2000-2000 (H), AF394217; WNV-CAR-HB6343-1989 (H), AF001558; WNV-ALG-ArDjanet-1968 (M), AF001567; WNV-SEN-AnD27875-1979 (primate, P), AF001569; WNV-CAR-ArB310-1967 (M), AF001566; WNV-I.C.-ArA3212-1981 (M), AF001561; KUNV-AUS-MRM61c-1960 (M), D00246; KUNV-AUS-Boort-1984 (E), AF196519; KUNV-AUS-P1553-1994 (M), AF196495; WNV-IND-G2266-1955 (M), AF196525; WNV-IND-804994-1980 (H), AF196526; WNV-IND-G16919-1955, AF205885; WNV-WENGLER, M12294; WNV-UGA-B956-1937 (H), AF394221; WNV-SEN-ArD78016-1990 (M), AF001556; WNV-UGA-Ent63134, AF001573; WNV-UGA-MP22-1959 (M), AF001562; WNV-RCA-AnB3507-1972 (B), AF001563; WNV-CAR-HB83P55-1983 (H), AF001557; WNV-RCA-ArB3573-1972 (M), AF001565; WNV-MAD-ArMg956-1986 (M), AF001564; WNV-KEN-Na1047 (M), AF001571; WNV-MAD-ArMg978-1988 (M), and AF001574.

 The extent to which this WNV genotype contributed to human disease in the 2000 epidemic remains undetermined. WNV-ISR-hISR2000 may have been carried into Israel by migrating birds from reservoirs in southeastern Europe or reservoirs in northeastern Africa, where a highly related virus was isolated in 1998 (WNV-KEN-KN3829-1998) (*18*). The 2000 Israel isolates in birds (and pigs, strains ISR-00GooMaS and ISR-00PigC) were different from the previous Israeli isolates (1997/98/99; strains ISR-97Goo1, ISR-98Goo1, ISR-IS98St1, ISR-99Goo, and ISR-99Gull [Figure]), but similar to the human isolate. Nonetheless, precedent exists for implicating more than one genotypic variant in a WNV outbreak. During the 1999 outbreak in Volgograd, Russia, two different genotypes were isolated: WNV-RUS-ASTR986-1999 (similar to 1997-98-99 Israeli and the WNV-NY1999 isolates, genotype lineage I subtype 2a) and WNV-VLG22889/WNV-RUS-VLG4-1999 (similar to WNV-ISR-hISR2000, subtype 2b [Figure]) (*19*). Indeed, even more divergent genotypes were identified during the 1996-97 WNV outbreak in Romania (WNV-RO97-50-1996 similar to WNV-ISR-hISR2000, genotype lineage I subtype 2b; WNV-RO96-1030-1996 and WNV-ROM96(0334)-1996, belonging to a different subtype, subtype 1 [Figure]) (*20*). The fact that no such divergence of genotypes of WNV isolates was observed during the 1999 New York epidemic (Figure) was interpreted as being compatible with a single, new introduction of this virus to the Western Hemisphere. While this manuscript was under review, another group reported WNV sequences from four patients of the 2000 Israel outbreak: two isolates most closely related to WNV-R097-50-1996 and two identical to the WNV-NY1999isolates (*21*). Analysis of additional isolates from the Israel 2000 and other outbreaks, including isolates obtained in 2000, 2001, and subsequent years in the USA, will be required to establish the extent to which avian migration and viral mutation contribute to the epidemiology of WNV-related disease.
